# Macroevolutionary diversification of glands for chemical communication in squamate reptiles

**DOI:** 10.1038/s41598-017-09083-7

**Published:** 2017-08-24

**Authors:** Roberto García-Roa, Manuel Jara, Simon Baeckens, Pilar López, Raoul Van Damme, José Martín, Daniel Pincheira-Donoso

**Affiliations:** 1 Department of Evolutionary Ecology, National Museum of Natural Sciences – Spanish Research Council (MNCN-CSIC), José Gutiérrez Abascal, 2, 28006 Madrid, Spain; 2 Laboratory of Evolutionary Ecology of Adaptations, School of Life Sciences, University of Lincoln, Joseph Banks Laboratories, Brayford Campus, Lincoln, LN6 7DL United Kingdom; 3 Department of Biology, Laboratory of Functional Morphology, University of Antwerp, Universiteitsplein 1, 2610 Wilrijk, Belgium

## Abstract

Chemical communication plays a central role in social, sexual and ecological interactions among animals. However, the macroevolutionary diversification of traits responsible for chemical signaling remains fundamentally unknown. Most research investigating evolutionary diversification of glands responsible for the production of chemical signals has focused on arthropods, while its study among vertebrates remains neglected. Using a global-scale dataset covering > 80% (7,904 species) of the living diversity of lizards and snakes (squamates), we investigate rates, trajectories and phylogenetic patterns of diversification of their follicular glands for chemical communication. We observed these glands in 13.66% of species, that their expression has varying phylogenetic signal among lineages, and that the crown squamate ancestor lacked follicular glands, which therefore originated and diversified subsequently during their evolutionary history. Additionally, our findings challenge the longstanding view that within squamates the Iguania are visually oriented while Scleroglossa are chemically-oriented, given that Iguania doubles Scleroglossa in the frequency of glands. Our phylogenetic analyses identified stabilizing selection as the best model describing follicular gland diversification, and revealed high rates of disparity. We provide the first global-scale analysis investigating the diversification of one of the main forms of communication among reptiles, presenting a macroevolutionary angle to questions traditionally explored at microevolutionary scale.

## Introduction

Communication plays a central role in ecological, social and sexual interactions among animals^1, 2^. Therefore, processes of diversification of signaling systems are often the outcome of complex evolutionary trajectories resulting from the interaction between natural and sexual selection^3^. A wide range of mostly visual, acoustic, and chemical signaling modes has proliferated across the animal tree of life^4, 5^. Among squamate reptiles (lizards and snakes), for example, while some lineages (*e.g*. anoles) exhibit strikingly colored ornaments, such as dewlaps^6^, other groups (*e.g*. geckos) have evolved the ability to influence the outcome of social interactions via vocalizations^7^. Indeed, these two channels of communication (visual and acoustic) have been the predominant focus of most research investigating dynamics of animal interactions mediated by the delivery of information via signals^1, 8^. Chemical communication, in contrast, has only recently seen a rather steep increase of interest in understanding how interactions via chemical cues and signals can underlie and influence the outcome of naturally- and sexually-selected interactions that cannot entirely be explained via visual and acoustic signals^9, 10^.

Following accelerated advances in the development of methodologies employed to analyze chemical signals^11^, the field of chemical ecology has rapidly expanded, and is increasingly contributing to our understanding of population and community dynamics mediated by communication^9, 12^. In fact, a number of studies conducted on invertebrates and vertebrates has revealed that the behavioral basis of multiple interactions depend primarily on chemical signals^9, 12–14^. Squamates embody a central example of how the study of chemical communication has influenced our understanding of animal interactions. For example, although the role of female mate choice in sexual competition has been shown to be widespread among animals^15, 16^, this form of sexual selection has been difficult to detect in reptiles, leading to the idea that competition over mates if fundamentally mediated by male-male contests^17^. However, emerging evidence suggest that female lizards can in fact choose males based on the chemical signals they produce^18–20^. Similar studies have shown the role for chemical signals in other forms of social interactions in these vertebrates, including territoriality^21, 22^, social recognition^23^ and conspecific assessment^24, 25^.

Even though reptiles deliver scents in multiple ways (e.g., via the skin, or feces)^13^, chemical communication among these organisms seems to be predominantly based on signals produced by follicular epidermal glands (FG)^26–28^. These glands are specialized tubular structures embedded in the dermis that discharge waxy secretions that are delivered into the external environment through epidermal pores^26, 29^. The numbers and body locations of FG differ widely across species and lineages^26^, varying from zero to ~130, while they can be situated around the cloaca (‘precloacal’), on the ventral surface of the thighs (‘femoral’), or in both regions^26^. In the majority of cases, these FG are restricted to, or show more complexity in males. Surprisingly, however, only a few studies restricted to specific lineages have investigated the evolution of these glands. Some studies have hypothesized that the wide interspecific diversity of FG could be the result of selective mechanisms mediating the enhancement of efficacy of the chemical signals as a function of the environments that species reside in^30–32^. Given the energetically costly demands associated with the development and functioning of these structures, selective pressures (e.g., allocation of energy, environmental factors) could shape adaptive variation of FG. On the other hand, a comparative study conducted in the hyper-diverse lizard genus *Liolaemus* revealed that, despite a previous conclusion of adaptive evolution^32^, the primary factor explaining patterns of FG numbers across lineages was shared ancestry^30^. Likewise, another study on lacertid lizards showed a marginal environmental effect on FG number and strong phylogenetic inertia^31^. However, and despite these phylogenetic effects within lizard families, FG have extensively evolved along the squamate phylogenetic history. Therefore, although these studies have shed some light on the factors influencing variation in FG, no research investigating the evolutionary trajectories and rates of these organs across a truly diverse range of lineages and areas of the world exists.

In this paper, we present the first global-scale study investigating the evolution of FG across the squamate tree of life. Based on an originally created dataset for 7,904 species and a molecular phylogeny containing 3,533 of the species for which FG data are available, we implement a model-selection approach aimed to quantitatively establish the evolutionary trajectories, tempo and mode of diversification of these organs.

## Results

### Diversity of follicular epidermal glands

**Figure 1:**
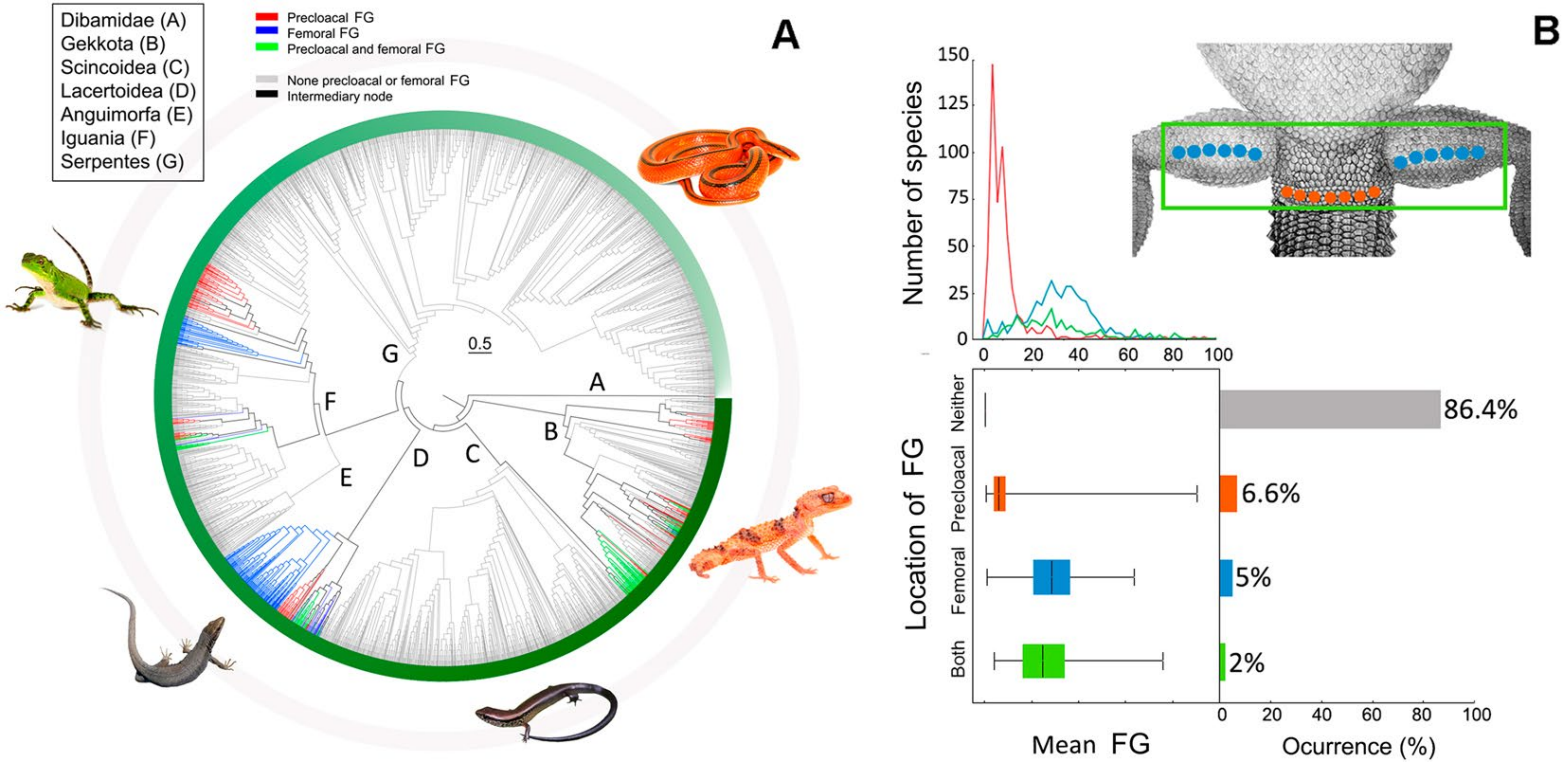
Overall distribution of number and location of follicular epidermal gland (FG) in Squamata. (**A**) Phylogenetic view of the FG location across Squamata phylogeny. The color of the branches indicates the absence or presence of FG, and their anatomical location (red: precloacal FG; blue: femoral FG; green: continuous row of FG from femoral to precloacal region; grey: No FG present at all; black: intermediate branches). The green band encircling the phylogeny merely indicates the direction of ancestry (scale at the top of the graph). Sample sizes: 3533 species. (**B**) Descriptive summary of presence, mean and location of FG in squamates. Top drawing shows potential locations of FG. The top graph shows the number of species (Y-axis) having particular numbers of FG (red: precloacal; blue: femoral; green: both locations). Box-plot graph illustrate different locations of FG (whiskers show max-min values). The histogram shows the percentage of species that had both locations of FG, femoral FG only, precloacal FG only, and no FG at all. In all cases color defines location of FG (red: precloacal FG; blue: femoral FG; green: continue row of FG from femoral to precloacal region). Photos: Roberto García-Roa (*Iberoacerta cyreni* and *Sphenomorphus cherriei*), James D. Emerson (*Oreocryptophis porphyraceus* and *Nephrurus wheeleri*) and Santiago Ron (*Iguana iguana*).

We found that FG are present in 13.66% of the 7,904 species in our dataset (Supplementary Table S1). After excluding snakes (which entirely lack FG), a total of 24.8% of the squamates have FG. These proportions vary across clades, FG being present in 35.2% of the Gekkota species, 26.82% of Iguania, and in 96.8% of Lacertoidea. In contrast, lower proportions of species with FG were found in Dibamidae (14.28%) and Scincoidae (1.11%) (Fig. 1B; Table 1). FG are entirely lacking from the clade Anguimorpha (173 species). Importantly, the proportion of species with FG is higher in Iguania (26.82%) than in Scleroglossa (which contains all other squamate lineages, with an 11.11%). The gecko *Mniarogekko chaoua* was identified as the species with the highest mean number of FG (95), while multiple species of different subclades presented only 1 or 2 of them. Regarding the location of FG, we found that 47.82% of species only have precloacal FG, 36.49% only femoral FG, and 15.69% have both types (Fig. 1B).

**Table 1:** Summary of information about the presence and number of follicular epidermal glands (FG) in squamates.

	Squamates (n = 7904)	Gekkota (n = 841)	Iguania (n = 1264)	Lacertoidea (n = 437)	Scincoidea (n = 1619)	Dibamidae (n = 7)
Number of FG	19.53 ± 0.49	21.05 ± 1.05	13.92 ± 0.74	28.86 ± 0.75	22.72 ± 2.43	4
Number of precloacal FG	8.51 ± 0.45	13.57 ± 1.13	6.64 ± 0.35	3.89 ± 0.18	0	4
Number of femoral FG	29.98 ± 0.61	25.68 ± 3.87	32.74 ± 1.24	29.83 ± 0.73	22.72 ± 2.43	0
Number of both FG	28.81 ± 1.28	33.22 ± 1.61	23.84 ± 2.64	21.22 ± 2.27	0	0
Species with FG	1077	296	338	423	18	1
Species with precloacal FG	515	178	238	97	0	0
Species with femoral FG	392	14	84	276	18	1
Species with both FG	170	104	16	50	0	0

The number of ‘precloacal FG’ (glands located on the edge of the cloacae), ‘femoral FG’ (glands on the ventral surface of the thighs) and ‘both FG’ (when a continuous row of glands expands from one hind limb to the other through the cloacae area) is presented as the average (±SE).

Analyses conducted on the variation of FG number across major squamates lineages (*i.e*. Gekkota, Iguania, Lacertoidea and Scincoidea) revealed that these structures differ significantly among groups (ANOVA, *F*_3,1074 _ =21.68, *P < *0.001). Subsequent pairwise comparisons between clades showed that differences were significant between Iguania and Lacertoidea (*Tukey’s* test: *P < *0.001), but not between Iguania and Gekkota (*P* = 0.46), and Iguania and Scincoidea (*P* = 0.09). Likewise, no differences were found between Gekkota and Lacertoidea (*P* = 0.46), Gekkota and Scincoidea (*P* = 0.97), nor Lacertoidea and Scincoidea (*P* = 0.99).

Analyses performed among species grouped according to whether they have precloacal, femoral or both forms of FG revealed significant differences in their mean number (phylANOVA; *F*_2,605_ = 172.58, *P* = 0.016). Species with precloacal FG have on average a lower number of these glands (mean = 8.51) compared to species with femoral FG (mean = 29.98; *P* = 0.024), or with glands in both locations (mean = 28.81; *P* = 0.034).

### Phylogenetic signal and ancestral state

Overall, the number of FG showed a moderate phylogenetic signal in Squamata, with a high *λ* and intermediate *K* (Table 2). A qualitatively similar degree of phylogenetic signal was observed in the subclades Gekkota and Lacertoidea. In contrast, *K*-values were very high in Iguania and especially in Scincoidea, meaning that species from these clades resemble each other more in their mean number of FG than expected under Brownian motion of evolution. The body location of FG (cloacal, femoral or both) was found to be highly phylogenetically conserved in squamates, and in lizards in particular (Table 2).

**Table 2:** Phylogenetical signals (Pagel’s λ and Blomberg’s K) and results of ancestral state reconstructions of follicular epidermal gland (FG) location, calculated for all squamates, and for lizards, and lizard subclades separately.

	FG number				Anatomical FG location						
	Phylogenetic signal				Phylogenetic signal	Ancestral state reconstruction					
	Blomberg’s K	P	Pagel λ	P	Pagel λ	Rate Index Estimate	SD	Scaled likelihoods at the root			
								Absent	Precloacal	Femoral	Both
Squamates	0.539	0.001	0.989	<0.001	0.999	0.0813	0.0068	0.997	<0.001	<0.001	<0.001
Lizards	0.572	0.001	0.978	<0.001	0.999	0.1146	0.0096	0.999	<0.001	<0.001	<0.001
Gekkota	0.407	0.001	0.998	<0.001	0.999	0.1668	0.0218	0.978	0.0210	<0.001	<0.001
Scincoidae	8.156	0.001	0.999	<0.001	0.999	0.0388	0.0195	0.207	0	0	0.792
Lacertoidea	0.44	0.001	0.846	<0.001	0.999	0.2273	0.0375	0.045	0.053	0.894	0.007
Iguania	1.99	0.001	0.981	<0.001	0.999	0.106	0.0173	0.87	0.002	0.126	0.003

#### Ancestral reconstructions

**Figure 2:**
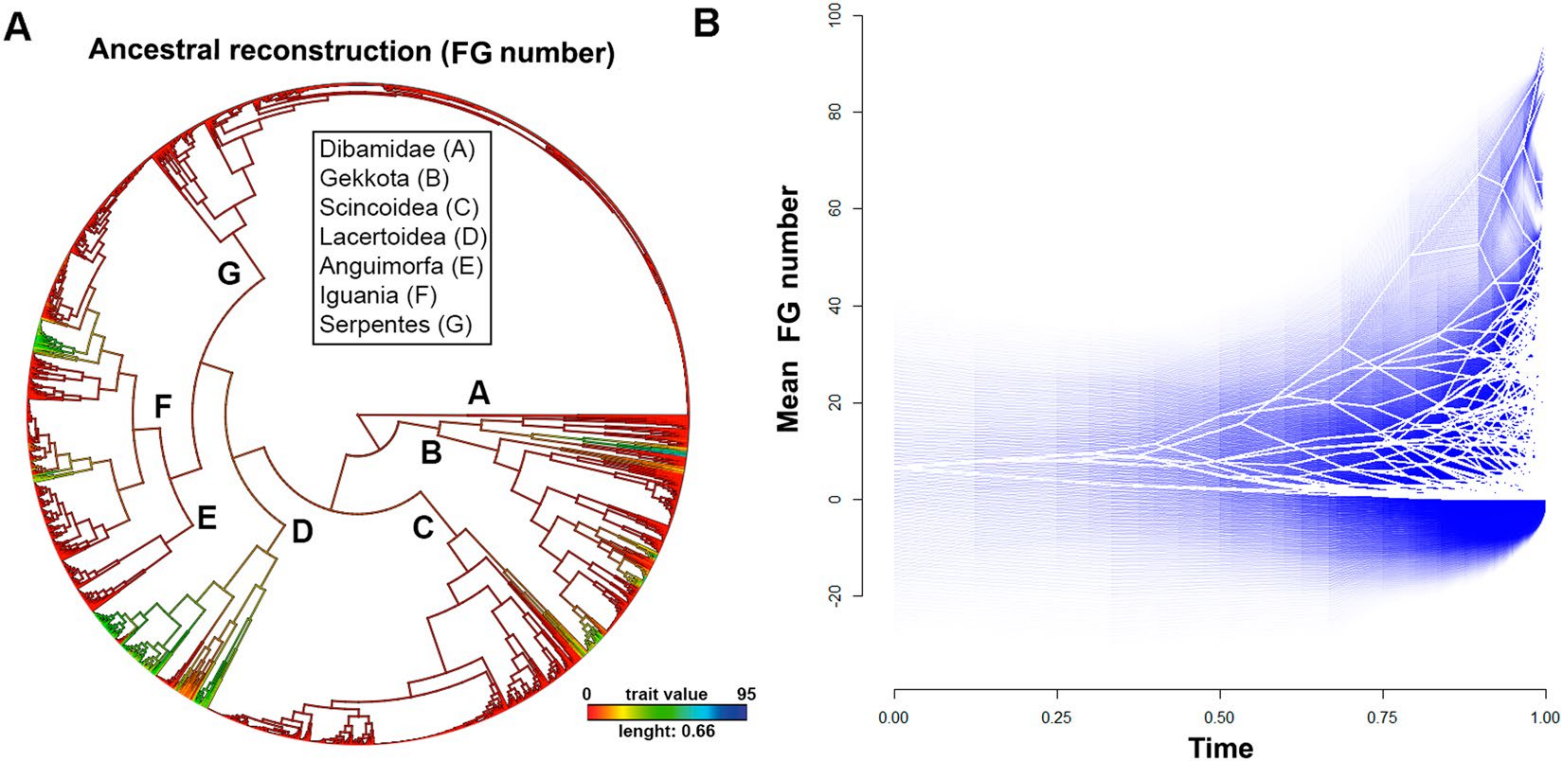
Diversification of follicular epidermal gland (FG) number across squamates. (**A**) Maximum likelihood ancestral character state reconstruction of FG number across Squamata phylogeny. (**B**) Projection of the Squamata phylogeny into a morphospace defined by relative time since the clades´ origin (X-axes) and FG number (Y-axis). Ancestral FG number is calculated using maximum likelihood. The increase of transparency of blue lines mirrors the degree of statistical uncertainty with 95% confidence interval.

**Figure 3:**
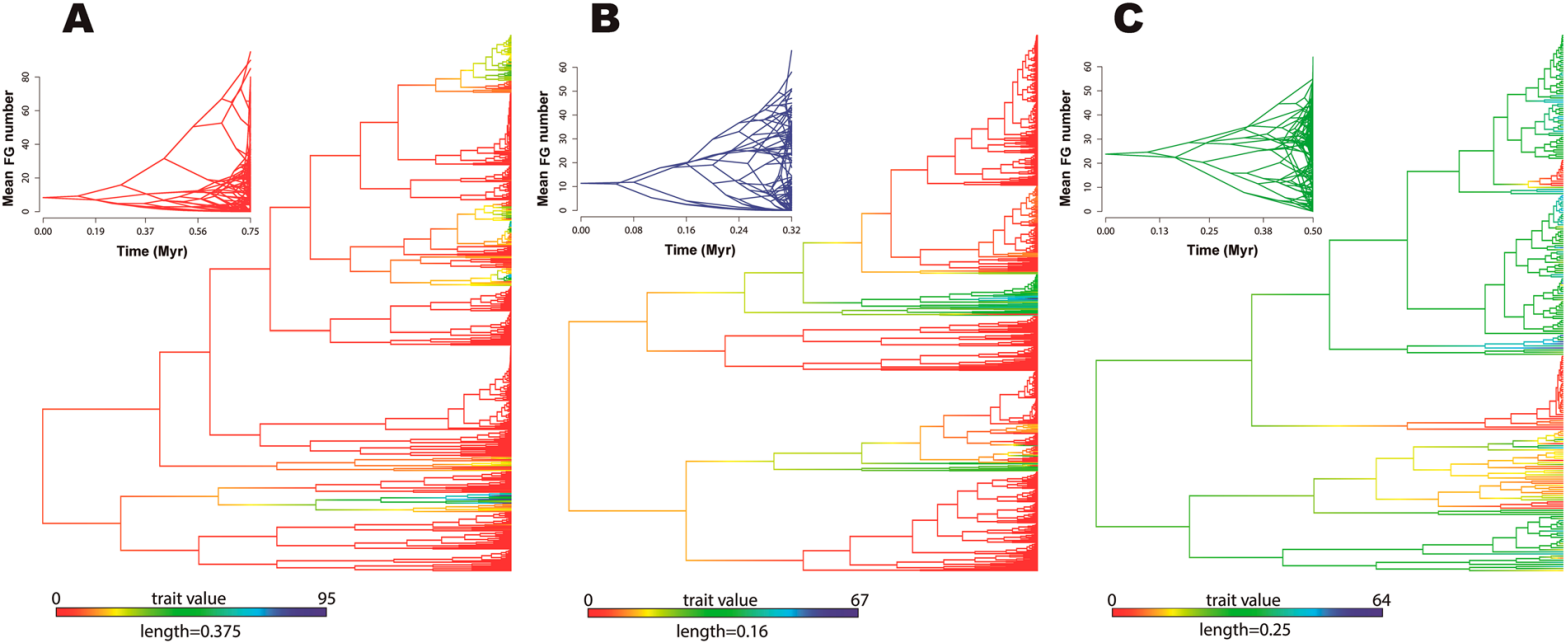
Ancestral character estimations and phenograms of the follicular epidermal gland (FG) in Gekkota, Iguania and Lacertoidea lineages. The phylogenetic trees (**A**: Gekkota; **B**: Iguania and **C**: Lacertoidea) reveal the maximum-likelihood phylogenetic ancestral character state reconstructions of FG number along the branches and nodes of the three lineages. Top tree of each phylogeny shows the morphospace´s projection defined by the relative time since the clades’ origin (X-axes) and pore number (Y-axis).

Ancestral state reconstructions revealed that the basal ancestor of modern squamates lacked FG (likelihood [LL] = 99.7%; Table 2 and Fig. 2A). The same observation was revealed for the ancestor of lizards (LL = 99.9%; Fig. 3), and for the subclade Gekkota in particular (LL = 99.78%). In addition, we observed a transition at the Scincoidea root from no FG into a state with a continuous row of FG (LL = 79.2%), and one with femoral FG at the Lacertoidea root (LL = 89.4%). Finally, in the Iguania phylogeny we observed at least one reversion from FG towards a total lack of FG (LL = 87%; Figs 2A and 3).

#### Macro-evolutionary patterns and models

Our model-selection analyses investigating macroevolutionary diversification dynamics of FG across squamates identified the Ornstein Uhlenbeck model (OU) as the best approximation describing the tempo and mode of evolution of these structures in squamates in general, as well as in Gekkota, Iguania and Lacertoidea when analyzed separately. The Delta, Brownian Motion (BM) and Early-Burst (EB) models were ranked in decreasing order based on their Akaike Information Criterion (AIC) values after the OU model (Table 3).

**Table 3:** Parameters and statistical fit of four models of evolutionary change used to describe the evolution of the number of epidermal glands in squamates.

Lineage	Model	Model parameters	β	LogL	AICc	ΔAICc
Squamata	BM	—	2075.41	−8861.71	17727.43	103.5
	OU	α = 2.71	2132.26	−8808.96	17623.93	0
	EB	α = −0.00	2075.48	−8861.71	17729.43	105.5
	Delta	δ = 2.99	698.39	−8835.35	17676.71	52.78
Gekkota	BM	—	8636.35	−1776.57	3557.17	58.39
	OU	α = 2.71	8835.83	−1746.36	3498.78	0
	EB	α = −0.00	8636.24	−1776.57	3559.2	60.42
	Delta	δ = 2.99	2911.82	−1758.08	3522.22	23.44
Iguania	BM	—	1296.99	−1897.94	3799.88	2.52
	OU	α = 2.51	1347.21	−1895.66	3797.36	0
	EB	α = −0.00	1296.97	−1897.93	3801.9	4.54
	Delta	δ = 2.01	661.82	−1896.04	3798.12	0.76
Lacertoidea	BM	—	9628.89	−1011.86	2027.77	19.35
	OU	α = 2.71	9825.2	−1001.16	2008.42	0
	EB	α = −0.00	9628.57	−1011.86	2029.782	21.362
	Delta	δ = 2.99	3261.19	−1002.27	2010.64	2.22

Data values are based on comparisons of four fitted evolutionary models: Brownian-motion (BM), Ornstein-Uhlenbeck (OU), Early-Burst (EB) and Delta. They were best-fitted based on bias corrected Akaike Information Criteria (AICc).

**Figure 4:**
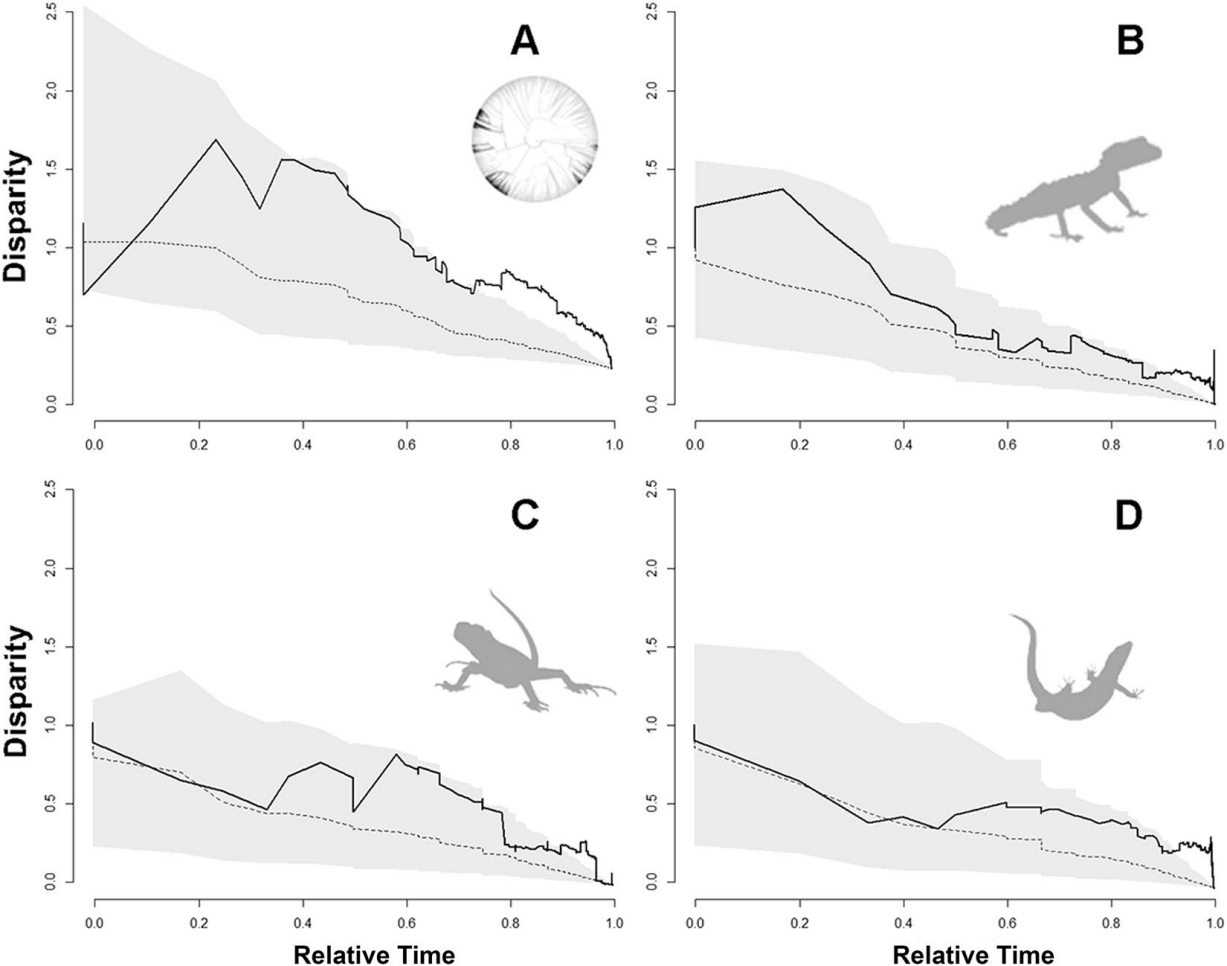
Mean disparity in follicular epidermal gland (FG) number through time (DTT) in squamates. The solid lines denote the actual relative disparity in FG number, while dotted line represents the expected values under Brownian Motion model of evolution based on 10 000 randomizations. The grey band shows the 95% confidence interval of DTT range. (**A**) Squamata; (**B**) Gekkota; (**C**) Iguania and (**D**) Lacertoidea.

Disparity-through-time analyses (DTT) performed on the squamate tree returned a positive morphological disparity index (MDI = 0.07), indicating high levels of subclade disparity: *i.e*. the subclades have diversified considerably and the ranges of their FG numbers overlap extensively (Figs 2B and 4). We also obtained positive relative disparity indices for Gekkota (MDI = 0.23), Iguania (MDI = 0.16) and Lacertoidea (MDI = 0.10), when analyzed separately (Fig. 4), which, again, indicates that most of the variation in FG numbers occurs within, not among subclades (Figs 2 and 3).

## Discussion

Our study provides the first global-scale analysis investigating the phenotypic diversity, phylogenetic distribution and evolutionary diversification dynamics of the FG used by squamate reptiles in chemical communication. Our results reveal that the degree of phylogenetic signal underlying variation in the number of FG is moderate across the squamate phylogeny. However, the strength of this phylogenetic signal varies among subclades, with some squamate lineages showing higher evolutionary lability than other clades. The diversification dynamics of FG numbers across lineages is described by a stabilizing selection model of evolution (OU). The evolution of relative disparity in FG numbers lies above the values expected from a Brownian motion model, which implies that subclades overlap with one another in FG morphospace, indicating a substantial degree of evolution of similar ‘gland strategies’ across different squamate clades. Our ancestral state reconstructions coupled with diversification analyses suggest that despite the observed phylogenetic signal, FG have extensively diversified among lizards over time and across lineages, after stemming from a basal ancestor that likely lacked these glands, and from which these structures emerged and disappeared in repeated evolutionary episodes during the squamate evolutionary history.

Squamate follicular glands play a paramount role in mediating and shaping conspecific and heterospecific interactions via the delivery of chemical signals for social and sexual communication [see reviews^13, 28, 33^]. However, most information about the evolution of these glands known so far only comes from two lizard groups, lacertids and *Liolaemus* ^30, 34, 35^. Indeed, patterns of morphological (*e.g*. tongue shape, number of sensory cells in their vomeronasal organ) and behavioral (number of average tongue-flicks and foraging mode) variation suggests that the evolution of chemo-sensation in squamates experienced a drastic episode of divergence early in the evolutionary history of this reptile group, followed by phylogenetic stability in the diversification of modes of chemical communication in the sublineages that subsequently originated from these crown ancestors^36–39^. Thus, Iguania is known to mostly be a ‘visually-oriented’ lineage, while the Scleroglossa are traditionally known to be ‘chemically-oriented’ organisms^36, 38^. Remarkably, however, our findings posit a challenge to this longstanding hypothesis, given that the proportion of species with FG is in fact much higher in Iguania than in Scleroglossa. Therefore, our global-scale analysis reveals the need to hypothesize that chemical communication is likely to have played a central role during the evolution of social and interspecific interactions within the cosmopolitan clade Iguania and not only Scleroglossa^30, 35, 39, 40^. Furthermore, some exceptionally-diverse lineages within Scleroglossa (*e.g*. skinks and snakes) have few or no species with FG (1.11% and 0, respectively), reinforcing the need to re-assess the currently accepted conclusions about the role of chemical communication in the global radiation of squamate reptiles as a whole (which accounts for >96% of living reptiles^41^). However, it is important to point-out that delivery of information via chemical signals is rarely (if ever) the result of one single source of structures that produce and deliver chemical signals into the environment. In fact, vertebrates in general have evolved multiple approaches to produce signals through the skin, feces, body fluids (*e.g*. saliva, urine), and vaginal secretions, among others. However, despite the wide diversity of systems for chemical signaling that have independently proliferated among different lineages of vertebrates, it is interesting to note that glands such as FG and the equivalent structures in a range of mammals show some important level of phenotypic conservatism within lineages. For example, while a range of studies have reported extensive variation in the chemical composition of FG signals across closely related species of lizards^32, 42^, our study confirms that there is a considerably degree of phylogenetic signal that underlies the expression of the glands’ structures across species. The same remains true for other distantly related lineages such as rodents. Therefore, although our findings reveal extreme diversification of FG across the squamate tree of life, it seems clear that selection operating on these structures is independent from selective pressures driving diversification of the chemical structure of the signals themselves.

Scincoidea and Iguania, in particular, were found to show phylogenetic conservatism in the variation of FG numbers across subclades, a finding that is consistence with a previous study^30^. In Gekkota and Lacertoidea, the phylogenetic signal is only moderate, which highlights the asymmetries in the strength of phylogenetic inertia and adaptive lability of chemical glands across the squamate tree of life. In fact, a recent study conducted on lacertid lizards revealed a moderate effect of phylogenetic ancestry shaping variation in FG, but also, a moderate predictability of variation in FG as a function of differential occupation of substrates among species^31^. In addition, our results identified stabilizing selection as the best approximation explaining the diversification of FG numbers, a finding that is further supported by the strong subclade overlap in morphospace also revealed by the DTT analysis. Therefore, and although shared ancestry remains an important factor underlying FG numbers, our results suggest that the number of these glands gravitates towards optimal values in both squamates as a whole, and in Gekkota, Iguania and Lacertoidea in particular.

Our analyses reveal that the ancestral state in squamates (and in lizards) is the lack of FG (see Table 1, for multiple ancestral states with and without FG across squamates). In view that the absence of FG seems to be the ancestral state for this clade, the subsequent appearance of these glands might have arisen in response to the need to engage in communication via an alternative signaling channel. Thus, given the high extent of diversity of signaling methods known among squamates (sounds, colours, behavioural displays, chemicals, among others), the differential degree of investment of each species into chemical signaling could condition the final expression (*i.e*. number and location) of the FG. For example, some lineages (*e.g*. snakes), in which the lack of FG is well known^13, 27^, are likely to have satisfied their communication demands via the specialization of alternative sources of chemical signals, such as the skin or feces. Indeed, multiple studies have shown the high physiological costs associated with the production and maintenance of systems for chemical communication in squamates^27, 33, 34^. Therefore, species with multimodal signaling systems (acoustic, visual or/and chemical), might be more constrained in the production and maintenance of these signaling traits than those that base their communication on one mode of communication only. In addition, to be dependent on other communication channels, FG are also closely interrelated with their chemical secretions. Accumulating evidence reveals the extraordinary qualitative and quantitative diversity of compounds present in these secretions^13, 43, 44^. Therefore, the production and maintenance of chemical signaling structures that improve the efficiency of the chemosensory system do not only depend on the FG, but also on the specific level of energetic allocation into the secretions^42^. Consequently, the evolution of chemical communication may be primarily targeted by selection operating on the efficiency of the signal itself rather than on the FG.

While ancestry is revealed as an important factor underlying the global patterns of variation in FG (including both number and location), the factors (*e.g*. environmental demands) underlying the observed tempo and mode of diversification presented by our study remain largely an open question. The strength of our global-scale study provides the first compelling overview that reinforces the view that different lineages have evolved different degrees of adaptive lability underlying the diversification of these glands. Some authors have suggested the hypothesis of between-channel compensation^31^ in which, given harsh environmental conditions, species might increase investment in additional or alternative signaling channels that are likely to promote changes in the evolutionary direction of the existing sensory channel (*e.g*. FG), leading to shifts in numbers, origins or losses of them. The field of chemical communication in reptiles offers a plethora of open questions and our study provides the fundamental empirical basis to guide the directions of further studies investigating the evolutionary dynamics of these interactions among one of the most diverse groups of tetrapods on Earth.

## Material and Methods

### Data Collection

We assembled a global dataset on the presence, number and location of FG for 7,904 species of squamates from the literature. These data cover 94% of all squamate families and over 80% of all species (see Table S1). To guarantee a comprehensive account of the phylogenetic distribution of the variation of these glands, and to inform the phylogenetic models about where the traits exist and where they have been lost, our data include species with and without them. Also, given that females of many species lack FG, we focused on males only. For each species, we obtained the mean pore number (*i.e*. pores on both left and right thigh) calculated from the average of multiple samples or as the midpoint between the minimum and maximum number of pores depending on the kind of information made available in the literature. When multiple independent sources provided information for the same species, we averaged data provided by all published sources for a species. FG were classified based on their location as ‘precloacal’ (located on the edge of the cloacae), ‘femoral’ (on the ventral surface of the thighs), both (when a continuous row of glands expands from one hind limb to the other through the cloacae area), or neither (glands are absent).

### Comparative analyses

In all our analyses, we used Pyron *et al*.’s^45^ molecular phylogenetic supertree for 4,161 squamate species. Of the 7,094 species for which we had FG data, we were able to include 3,533 species into this tree.

To assess evolutionary patterns in the number and location of FG, we first tested if the average number of FG differed depending on their anatomical location, using phylogenetic analyses of variance (‘phylANOVA’)^46^. We then examined potential differences in FG number among the four main taxa where FG are known to be present (*i.e*. Gekkota, Iguania, Lacertoidea and Scincoidea) based on non-phylogenetic GLMs. For these analyses, we deliberately excluded the Dibamidae family given that this small family of squamates has only one species with FG. Pairwise comparisons were based on Tukey’s HSD tests in all cases^47^. All statistical tests were performed using R 3.2.2 and SPSS 20.0.0 software.

### Phylogenetic signal and ancestral state

We estimated the phylogenetic signal for FG number and location in all squamates. Generally, phylogenetic signal is recognized to be the tendency for related species to resemble one another for a specific trait, and Pagel’s *λ* and Blomberg *K* are two quantitative measure of this pattern^48, 49^. A *λ*-value close to 0 indicates no phylogenetic structure in the trait, whereas a *λ*-value close to 1 indicates an increasingly stronger effect of shared ancestry on the expression of the trait across the species in the tree^48^. A *K-*value lower than 1 implies that relatives resemble each other less than expected under Brownian motion evolution along the hypothesized tree, whereas K > 1 implies that closely related species are more similar than expected under Brownian motion evolution. The signal of the continuous variables was assessed using Pagel’s λ and Blomberg’s K (with nsim = 1,000) and the ‘phylosig’ function in the ‘phytools’ package^46^. For the discrete variable ‘anatomical location of the pores’, we calculated Pagel’s λ only, using the ‘fitDiscrete function’ in the ‘geiger’ package^50^.

Additionally, we used ancestral character state reconstructions (‘ace’ function in the ‘ape’ package^51^) to quantitatively estimate ancestral states of FG location at the root of the Squamata tree, and at specific internal nodes of the different lineages within Squamata (*i.e*. Sauria, Gekkota, Iguania, Lacertoidea and Scincoidea). Finally, we employed the approach implemented by Revell and Freckleton^52^ to visualize the dynamic trajectories of ancestral states of FG numbers along branches of the phylogenic tree. These analyses use a Maximum-Likelihood approach to reconstruct ancestral states.

### Modelling FG evolution

To examine the diversification of FG number across Squamata, we used two approaches aimed to elucidate both global and lineage-specific macro-evolutionary diversification dynamics. We firstly analyzed the whole global dataset and then, we focused on the three lizard lineages with the largest number of species with FG in our dataset (*i.e*. Gekkota, Iguania and Lacertoidea). We fitted four alternative evolutionary models that describe different regimes of phenotypic evolution: i) the Brownian-motion model (BM) according to which evolution proceeds as a random walk through trait space; the expected phenotypic difference between two taxa grows proportional to the time since their divergence^53^; ii) the Ornstein-Uhlenbeck model (OU) which describes the evolution of traits under stabilizing selection – it modifies the BM model by considering one or several adaptive optima to which trait evolution is attracted as diversification proceeds during the phylogenetic history of the lineage^54^; iii) the Early-Burst model (EB) or ‘accelerating-decelerating model’, which assumes that species evolve under a density-dependent availability of niche space, predicting rapid early diversification followed by decreases in evolutionary rates as a result of saturation of niche space over time^55–57^; iv) the Delta model which allows the evolutionary rate to either decrease (δ < 1) or increase (δ > 1) through time, from root to tips. In a model with δ < 1, the trait changes rapidly early in the history of a clade and then slows down through time. If δ > 1, trait change accelerates through time^48, 53^. We performed the comparisons of goodness of fit for these four models based on Akaike Information Criterion (AIC)^58, 59^. We use the bias-corrected version of AIC (AICc) to determine the ΔAICc values, which result from the difference between the lowest AICc and the AICc of each alternative model. Therefore, the best-fit model has ΔAICc = 0^53, 60^. All these analyses were conducted using the ‘geiger’ package in R^50^.

We further investigated the disparity of the FG number over time. We performed disparity-through-time analyses (DTT) by plotting fluctuations in average relative disparity over time^61–63^. In such analyses, the mean disparity in the trait (here: FG number) at any node along the clades’ history is compared to the disparity under the null model of Brownian motion (estimated by the mean of 10,000 simulations). The average of these two values (*i.e*. FG number disparity from dataset and simulated data from BM) are plotted against node age to obtain the morphological disparity index (MDI)^53, 62, 64^. Thus, values below zero (*i.e*. those values lower than expected under BM model) indicate that most of disparity is among subclades, which are distributed in smaller and isolated morphospace regions. Instead, MDI values above zero mean that disparity among subclades is highly overlapped in the morphospace^53^. We carried out DTT analyses using the R package ‘geiger’^50^. Subsequently, we finally built a “traitgram” plotting the Squamata phylogeny onto the FG number morphospace over time since the root origin. The resulting projection is based on ancestral node estimations using maximum likelihood approaches^65^. These analyses were performed using the R package ‘phytools’^46^.

## Electronic supplementary material

Supplementary information

## References

[1] Smith, W. J. Animal communication and the study of cognition. In *Cognitive ethology: The minds of other animals*. (ed. Ristau, C.A.) 209-230 (Hillsdale, N. J.: Lawrence Erlbaum Association, 2013).

[2] Maynard Smith, J. & Harper, D. Animal signals: Oxford series in ecology and evolution. *Oxf. Univ. Press, NY*, 1–166 (2003).

[3] Searcy, W. A. & Nowicki, S. *The evolution of animal communication: reliability and deception in signaling systems*. (Princeton University Press, 2005).

[4] Cornwallis CK, Uller T (2010). Towards an evolutionary ecology of sexual traits. Trends Ecol. Evol..

[5] Bro-Jørgensen J (2010). Dynamics of multiple signalling systems: animal communication in a world in flux. Trends Ecol. Evol..

[6] Ng J, Landeen EL, Logsdon RM, Glor RE (2013). Correlation between Anolis lizard dewlap phenotype and environmental variation indicates adaptive divergence of a signal important to sexual selection and species recognition. Evolution.

[7] Yu X (2011). Geographic variation in the advertisement calls of *Gekko gecko* in relation to variations in morphological features: implications for regional population differentiation. Ethol. Ecol. Evol..

[8] Bradbury, J. W. & Vehrencamp, S. L. *Principles of animal communication*. Second edn. (2011).

[9] Wyatt, T. D. Pheromones and animal behavior: chemical signals and signatures. (Cambridge University Press, 2014).

[10] Wyatt TD (2010). Pheromones and signature mixtures: defining species-wide signals and variable cues for identity in both invertebrates and vertebrates. J. Comp. Physiol. A.

[11] Touhara, K. Pheromone signaling: methods and protocols. (Humana Press, 2013).

[12] Apps PJ, Weldon PJ, Kramer M (2015). Chemical signals in terrestrial vertebrates: search for design features. Nat. Prod. Rep..

[13] Weldon PJ, Flachsbarth B, Schulz S (2008). Natural products from the integument of nonavian reptiles. Nat. Prod. Rep..

[14] Johnston, R. & del Barco-Trillo, J. Communication by chemical signals: behavior, social recognition, hormones and the role of the vomeronasal and olfactory systems. *Hormones, brain and behavior* (eds Pfaff, D. W., Arnold, A. P., Etgen, A. M., Rubin, R. T. & Fahrbach, S. E.), 395–440 (2009).

[15] Andersson, M. B. *Sexual selection*. (Princeton University Press, 1994).

[16] Jennions MD, Petrie M (1997). Variation in mate choice and mating preferences: a review of causes and consequences. Biol. Rev..

[17] Olsson, M., Madsen, T. & Møller, A. In *13: Sexual selection and sperm competition in reptiles*. 503–577 (Academic Press San Diego, 1998).

[18] Martín J, López P (2006). Links between male quality, male chemical signals, and female mate choice in Iberian rock lizards. Funct. Ecol..

[19] López P, Martín J (2005). Female Iberian wall lizards prefer male scents that signal a better cell-mediated immune response. Biol. Lett..

[20] Martín J, López P (2000). Chemoreception, symmetry and mate choice in lizards. P. Roy. Soc. Lond. B Bio. Sci..

[21] Kratochvíl L, Frynta D (2002). Body size, male combat and the evolution of sexual dimorphism in eublepharid geckos (Squamata: Eublepharidae). Biol. J. Linn. Soc..

[22] Carazo P, Font E, Desfilis E (2008). Beyond ‘nasty neighbours’ and ‘dear enemies’? Individual recognition by scent marks in a lizard (*Podarcis hispanica*). Anim. Behav..

[23] Labra A, Niemeyer HM (1999). Intraspecific chemical recognition in the lizard *Liolaemus tenuis*. J. Chem. Ecol..

[24] Carazo P, Font E, Desfilis E (2007). Chemosensory assessment of rival competitive ability and scent-mark function in a lizard. Podarcis hispanica. Anim. Behav..

[25] Martín J, López P (2006). Vitamin D supplementation increases the attractiveness of males’ scent for female Iberian rock lizards. P. Roy. Soc. Lond. B Bio. Sci..

[26] Mayerl C, Baeckens S, Van Damme R (2015). Evolution and role of the follicular epidermal gland system in non-ophidian squamates. Amphibia-Reptilia.

[27] Mason RT, Parker MR (2010). Social behavior and pheromonal communication in reptiles. J. Comp. Physiol. A.

[28] Martín, J. & López, P. In *Reproductive Biology and Phylogeny of Lizards and Tuatara* (eds Rheubert, J. L., Siegel, D. S. & Trauth, S. E.) 43–75 (CRC Press, Boca Raton, Florida, 2014).

[29] Valdecantos S, Martínez V, Labra A (2014). Comparative morphology of Liolaemus lizards precloacal glands. Acta Herpetol..

[30] Pincheira-Donoso D, Hodgson DJ, Tregenza T (2008). Comparative evidence for strong phylogenetic inertia in precloacal signalling glands in a species-rich lizard clade. Evol. Ecol. Res..

[31] Baeckens S, Edwards S, Huyghe K, Van Damme R (2015). Chemical signalling in lizards: an interspecific comparison of femoral pore numbers in Lacertidae. Biol. J. Linn. Soc..

[32] Escobar CA, Labra A, Niemeyer HM (2001). Chemical composition of precloacal secretions of Liolaemus lizards. J. Chem. Ecol..

[33] Houck LD (2009). Pheromone communication in amphibians and reptiles. Annu. Rev. Physiol..

[34] Martín J, López P (2015). Condition-dependent chemosignals in reproductive behavior of lizards. Horm. Behav..

[35] Labra A (2011). Chemical stimuli and species recognition in Liolaemus lizards. J. Zool..

[36] Schwenk K (1993). The evolution of chemoreception in squamate reptiles: a phylogenetic approach. Brain, Behav. Evol..

[37] Schwenk K (1994). Comparative biology and the importance of cladistic classification: a case study from the sensory biology of squamate reptiles. Biol. J. Linn. Soc..

[38] Schwenk K (1995). Of tongues and noses: chemoreception in lizards and snakes. Trends Ecol. Evol..

[39] Vidal N, Hedges SB (2009). The molecular evolutionary tree of lizards, snakes, and amphisbaenians. C. R. Biol..

[40] Baeckens S, Driessens T, Van Damme R (2016). Intersexual chemo-sensation in a “visually-oriented” lizard. Anolis sagrei. PeerJ.

[41] Pincheira-Donoso D, Bauer AM, Meiri S, Uetz P (2013). Global taxonomic diversity of living reptiles. PLoS One.

[42] García‐Roa R, Jara M, López P, Martín J, Pincheira‐Donoso D (2017). Heterogeneous tempo and mode of evolutionary diversification of compounds in lizard chemical signals. Ecol. Evol..

[43] García-Roa R, Carreira S, López P, Martín J (2016). Genders matters: Sexual differences in chemical signals of *Liolaemus wiegmannii* lizards (Iguania, Liolaemidae). Biochem. Syst. Ecol..

[44] Runemark A, Gabirot M, Svensson E (2011). Population divergence in chemical signals and the potential for premating isolation between islet‐and mainland populations of the Skyros wall lizard (*Podarcis gaigeae*). J. Evol. Biol..

[45] Pyron RA, Burbrink FT, Wiens JJ (2013). A phylogeny and revised classification of Squamata, including 4161 species of lizards and snakes. BMC Evol. Biol..

[46] Revell L (2012). J. phytools: an R package for phylogenetic comparative biology (and other things). Methods Ecol. Evol..

[47] Sokal, R. & Rohlf, F. Biometry (3rd edn). *WH Freman and company: New York* (1995).

[48] Pagel M (1999). Inferring the historical patterns of biological evolution. Nature.

[49] Blomberg SP, Garland T, Ives AR (2003). Testing for phylogenetic signal in comparative data: behavioral traits are more labile. Evolution.

[50] Harmon LJ, Weir JT, Brock CD, Glor RE, Challenger W (2008). GEIGER: investigating evolutionary radiations. Bioinformatics.

[51] Paradis E, Claude J, Strimmer K (2004). APE: analyses of phylogenetics and evolution in R language. Bioinformatics.

[52] Revell LJ, Freckleton R (2013). Two new graphical methods for mapping trait evolution on phylogenies. Methods Ecol. Evol..

[53] Pincheira-Donoso D, Harvey LP, Ruta M (2015). What defines an adaptive radiation? Macroevolutionary diversification dynamics of an exceptionally species-rich continental lizard radiation. BMC Evol. Biol..

[54] Hansen, T. F. Stabilizing selection and the comparative analysis of adaptation. *Evolution*, 1341–1351 (1997). 10.1111/j.1558-5646.1997.tb01457.x 28568616

[55] Harmon LJ (2010). Early bursts of body size and shape evolution are rare in comparative data. Evolution.

[56] Blomberg SP, Garland T, Ives AR, Crespi B (2003). Testing for phylogenetic signal in comparative data: behavioral traits are more labile. Evolution.

[57] Paleo‐López R (2016). A phylogenetic analysis of macroevolutionary patterns in fermentative yeasts. Ecol. Evol..

[58] Akaike H (1974). A new look at the statistical model identification. Automatic Control, IEEE Transactions on.

[59] Akaike, H. In *Selected Papers of Hirotugu Akaike* 215–222 (Springer, 1998).

[60] Burnham, K. P. & Anderson, D. R. *Model selection and multimodel inference: a practical information-theoretic approach*. (Springer Science & Business Media, 2002).

[61] Frédérich B, Sorenson L, Santini F, Slater GJ, Alfaro ME (2013). Iterative ecological radiation and convergence during the evolutionary history of damselfishes (Pomacentridae). Am. Nat..

[62] Harmon LJ, Schulte J, Larson A, Losos JB (2003). Tempo and mode of evolutionary radiation in Iguanian lizards. Science.

[63] Ingram T (2015). Diversification of body shape in Sebastes rockfishes of the north‐east Pacific. Biol. J. Linn. Soc..

[64] Astudillo-Clavijo V, Arbour JH, Lopez-Fernandez H (2015). Selection towards different adaptive optima drove the early diversification of locomotor phenotypes in the radiation of Neotropical geophagine cichlids. BMC Evol. Biol..

[65] Ruta M, Wagner PJ, Coates MI (2006). Evolutionary patterns in early tetrapods. I. Rapid initial diversification followed by decrease in rates of character change. P. Roy. Soc. Lond. B Bio. Sci..

